# Comparison of Colorimetric Assays with Quantitative Amino Acid Analysis for Protein Quantification of Generalized Modules for Membrane Antigens (GMMA)

**DOI:** 10.1007/s12033-014-9804-7

**Published:** 2014-09-16

**Authors:** Omar Rossi, Luana Maggiore, Francesca Necchi, Oliver Koeberling, Calman A. MacLennan, Allan Saul, Christiane Gerke

**Affiliations:** Novartis Vaccines Institute for Global Health, Via Fiorentina 1, 53100 Siena, Italy

**Keywords:** Protein quantification, Generalized Modules for Membrane Antigens (GMMA), Outer Membrane Vesicles (OMV), Quantitative amino acid analysis, Colorimetric protein assay, Lowry assay, Bradford assay, Non-Interfering assay

## Abstract

Genetically induced outer membrane particles from Gram-negative bacteria, called Generalized Modules for Membrane Antigens (GMMA), are being investigated as vaccines. Rapid methods are required for estimating the protein content for in-process assays during production. Since GMMA are complex biological structures containing lipid and polysaccharide as well as protein, protein determinations are not necessarily straightforward. We compared protein quantification by Bradford, Lowry, and Non-Interfering assays using bovine serum albumin (BSA) as standard with quantitative amino acid (AA) analysis, the most accurate currently available method for protein quantification. The Lowry assay has the lowest inter- and intra-assay variation and gives the best linearity between protein amount and absorbance. In all three assays, the color yield (optical density per mass of protein) of GMMA was markedly different from that of BSA with a ratio of approximately 4 for the Bradford assay, and highly variable between different GMMA; and approximately 0.7 for the Lowry and Non-Interfering assays, highlighting the need for calibrating the standard used in the colorimetric assay against GMMA quantified by AA analysis. In terms of a combination of ease, reproducibility, and proportionality of protein measurement, and comparability between samples, the Lowry assay was superior to Bradford and Non-Interfering assays for GMMA quantification.

## Introduction

Gram-negative bacteria naturally shed outer membrane particles from their surface. These are small spherical structures that maintain the structure, composition, and orientation of the outer membrane, and also contain soluble periplasmic proteins entrapped in the lumen [1, 2]. Outer membrane particles constitute an attractive technology as new vaccines because they represent the envelope of Gram-negative bacteria and thus comprise many of the components that are recognized by the immune system. However, their yield is usually too low for practical production. A high-yield production process using genetic modification of the parent bacteria in order to induce high levels of shedding, coupled with efficient methods for production and purification, has been developed for application to vaccinology. The resulting outer membrane particles are called Generalized Modules for Membrane Antigens (GMMA) [3–5]. A GMMA vaccine produced and purified from a *Shigella* strain genetically engineered to increase their release by using a *tolR* mutation [3] is currently in Phase I clinical trials. The GMMA approach is also being evaluated for other Gram-negative bacteria, including *Neisseria meningitidis* [4].

Establishing a consistent and reproducible method for the quantification of GMMA is important for their functional characterization and for vaccine production. GMMA could be conveniently quantified based on protein amount, although other assays, e.g., for sugar or lipid content, may be appropriate for specific vaccines. Even in these cases, since protein assays are usually simpler to perform, for in-process assays, e.g., during purification or formulation, measuring protein content is preferred.

Quantitative amino acid (AA) analysis is a primary method for protein quantification [6], because it is not affected by protein composition and does not need a calibration curve with a standard protein. Routine analysis slightly underestimates the actual concentration due to the loss of cysteine and tryptophan residues during the analysis, but this difference is usually marginal and can be corrected in a more detailed analysis [7]. However, analysis based on AA content is laborious and expensive and, therefore, not suitable for in-process assays. For this reason, secondary colorimetric assays, using a standard protein, are usually preferred and different colorimetric assays have been used by various groups for measuring protein content in naturally shed outer membrane blebs [3, 8, 9]. However, previous studies comparing colorimetric assays (reviewed in [10]), or the results of colorimetric assays with AA quantification [11], highlighted the variability of the results from colorimetric assays since the color yield, the optical density (OD) obtained in the reaction per mass of protein, depends on the protein composition and the sequence of individual proteins [10].

In order to determine which colorimetric assay provides the best reproducibility, range, proportionality of the results to AA analysis, and ease of procedure for the measurement of protein content of GMMA, we compared Bradford [12], Lowry [13], and Non-Interfering (NI) (Geno Technology, Inc., St. Louis, MO, USA) protein assays using GMMA produced from different bacterial lines (*Shigella*, *Salmonella,* and *Neisseria*). The Bicinchoninic Acid (BCA) assay was not tested due to the presence of lipids in GMMA and the interference of lipids with the BCA assay [14]. GMMA protein concentration was evaluated using their AA content as the primary standard compared to bovine serum albumin (BSA) as a secondary standard, taking into account the relative color yield (the ratio of the color yield of GMMA as determined by AA content to the color yield of BSA). GMMA from parent bacteria with different genetic and phenotypic backgrounds (e.g., the presence/absence of O antigen, OAg) were studied.

## Materials and Methods

### Bacterial Strains

Mutant strains generated from *Shigella sonnei* 53G [15], *Shigella flexneri* 2a 2457T [16], *Salmonella enterica* serovar Typhimurium D23580 [17], and *Neisseria meningitidis* serogroup W [18] were used in this study and their identifications and descriptions are listed in Table 1.

**Table 1:** Strains used in this study with genotype and phenotype description

Strain name	Genotype	Phenotype	Reference
*Ss* −p ΔOAg	*S. sonnei* −pSS Δ*tolR::kan*	Increased GMMA release (Δ*tolR*); virulence plasmid absent; OAg-deficient (as OAg is encoded on pSS), nicotinic acid auxotroph	[3]
*Ss* +p ΔOAg	*S. sonnei* +pSS Δ*tolR::kan* Δ*wbg::nadAnadB*	Increased GMMA release (Δ*tolR*); OAg-deficient (Δ*wbg*); virulence plasmid present, nicotinic acid prototroph (*nadAnadB*)	Gerke C., unpublished work
*Ss* +p	*S. sonnei* +pSS Δ*tolR::kan* Δ*virG::nadAnadB*	Increased GMMA release (Δ*tolR*); virulence plasmid present, nicotinic acid prototroph (*nadAnadB*)	Gerke C., unpublished work
*Sf*2a +p ΔOAg	*S. flexneri* 2a +pINV Δ*tolR::kan* Δ*rfbG::erm*	Increased GMMA release (Δ*tolR*); OAg-deficient (Δ*rfbG*); virulence plasmid present	[5]
*Sf*2a +p	*S. flexneri* 2a +pINV Δ*tolR::kan*	Increased GMMA release (Δ*tolR*); virulence plasmid present	[5]
*Sf*2a −p ΔOAg	*S. flexneri* 2a −p INV Δ*tolR::kan* Δ*rfbG::erm*	Increased GMMA release (Δ*tolR*); OAg-deficient (Δ*rfbG*); virulence plasmid absent	[5]
*Salmonella*	*Salmonella enterica* Typhimurium Δ*tolR::cat* Δ*wbaP::kan*	Increased GMMA release (Δ*tolR*); OAg-deficient (Δ*wbaP*)	Necchi F., unpublished work
*Neisseria*	*Neisseria meningitidis* Δ*gna33::erm* Δ*cps::cat* Δ*lpxL1::kan*	Increased GMMA release (Δ*gna33*); reduction of reactogenicity (Δ*lpxL1*); capsule-deficient (Δ*cps*)	[4]

### Bacterial Growth

#### *Shigella*

*Shigella* *flexneri* 2a mutants were grown in yeast extract medium (HTMC [3]) with 200 µM 2,2-dipyridyl to induce overexpression of iron-regulated proteins. *Shigella* *sonnei* strains were grown in *Shigella sonnei* defined medium (SSDM [3]) with the exception that nicotinic acid was added for growth of nicotinic acid auxotroph *Ss* −p ΔOAg only, and was omitted for growth of nicotinic acid prototroph *Ss* +p ΔOAg and *Ss* +p. If required, kanamycin 30 µg/mL or erythromycin 100 µg/mL were added. Strains were grown at 37 °C. For GMMA production, overnight starter cultures of *Shigella* mutants were grown from glycerol stocks in 5 mL of media with selective antibiotics as appropriate. 300 mL of medium without antibiotics was inoculated with starter culture to an OD measured at 600 nm wavelength, OD_600_, of 0.05 and incubated for approximately 15 h until stationary phase.

#### *Salmonella*

Overnight cultures were grown in 5 mL Luria–Bertani (LB) medium from single colonies of *Salmonella* Typhimurium Δ*tolR* ΔOAg at 37 °C in the presence of chloramphenicol 20 µg/mL and kanamycin 30 µg/mL. The starter culture was used to inoculate 60 mL LB to OD_600_ 0.05, and the culture was incubated at 37 °C for approximately 9 h until stationary phase.

#### *Neisseria*

7 mL of a modified version of a defined medium described previously [19] was inoculated with single colonies of *N. meningitidis* Δ*gna33* Δ*cps* Δ*lpxL1* [4] to OD_600_ 0.15–0.17 and incubated at 37 °C, 5 % CO_2_ to mid log phase (OD_600_ 0.6). The starter culture was used to inoculate 50 mL medium to OD_600_ 0.05 and the culture was incubated for approximately 9 h at 37 °C, 5 % CO_2_ until stationary phase.

### Purification of GMMA

Cells were harvested by centrifugation (5,000×*g*, 30 min, 4 °C) and GMMA-containing culture supernatants were filtered through a 0.22 µm pore-size Stericup (Millipore, Billerica, MA, USA). If required, supernatants were concentrated to a volume of approximately 60 mL. GMMA were collected by ultracentrifugation (186,000×*g*, 2 h, 4 °C). The pellet was washed once with PBS buffer, resuspended in PBS, and passed through a 0.22-µm-filter [4, 5].

### Protein Quantification of GMMA

Before protein quantification, GMMA were solubilized using guanidine hydrochloride (Bradford assay), or SDS (Lowry assay), or denatured by protein precipitation (Non-Interfering assay), in order to dissolve their particulate nature and ensure full accessibility of all proteins.

#### Bradford

Bradford protein quantifications were performed using Bradford Protein Assay (Bio-Rad, Hercules, CA, USA). GMMA samples were diluted 1:2 with 6.0 M guanidine hydrochloride pH 7.8 and boiled for 10 min with occasional vortexing [3]. Different quantities of GMMA were adjusted to a final volume of 200 µL with water. 800 µL of Bradford reagent was added to each sample, and absorbance at 595 nm was measured after 30 min.

#### Lowry

For Lowry protein quantification, the Detergent Compatible (DC) Protein assay (Bio-Rad) was used. Different quantities of GMMA were adjusted to a final volume of 25 µL with water. 125 µL of freshly prepared reagent A′ (20 µL of reagent S, containing 5–10 % SDS, added to every mL of reagent A) was added and samples were vortexed. 1 mL of reagent B was added, and absorbance at 750 nm was read after 15 min.

#### Non-Interfering Protein Assay (NI Assay)

We used NI™ (Non-Interfering™) Protein Assay (G-Biosciences, St. Louis, MO, USA, a brand name of Geno Technology) and performed the assays according to the manufacturer’s instructions (NI manual). Briefly: different quantities of GMMA were adjusted to a final volume of 25 µL with water, 500 µL of Universal Protein Precipitation Agent (UPPA) I was added and vortexed after 2 min; 500 µL of UPPA II was added, vortexed, and centrifuged for 5 min at 10,000×*g* and supernatants discarded by inverting the tubes. The tubes were centrifuged again for 5 min at 10,000×*g*, supernatants were discarded with a pipette, and 500 µL of reagent 1 (100 µL of copper solution plus 400 µL of water) was added to each tube, and pellets were resuspended by vortexing. Finally, 1 mL of reagent 2 (1 part of Color reagent B added to every 100 parts of Color reagent A) was added, and the absorbance at 480 nm was read after 15 min.

Plastic cuvettes were used by default for all assays (less expensive and disposable). However, we found that they introduced certain variability when reading at 750 nm, and thus we used Quartz cuvettes for the defining the intra-assay variation in the Lowry assay.

Standard curves for the assays were prepared with BSA (Pierce, Rockford, IL, USA) in the range of 1–10 μg/assay for Bradford assay and 4–50 μg/assay for both Lowry and Non-Interfering assays according to the manufacturers’ specifications for the different assays. For Lowry and NI assay, the standard points were treated the same way as the GMMA samples. In the Bradford assay, treatment of BSA with guanidine hydrochloride did not have an impact on the absorbance of the BSA sample (data not shown) and thus this step was omitted. The standard curves were run in duplicate for each assay.

### Quantitative Amino Acid Analysis

Quantitative AA analysis of GMMA was performed by Alta Bioscience Ltd (Birmingham, UK) using an ion exchange separation of AA followed by post column detection with ninhydrin [7]. Stabilization of tryptophan and cysteine/cystine prior to acid hydrolysis was not performed, and thus these AAs were partially or totally degraded during the hydrolysis.

### Determination of BSA Equivalent, Color Yield Factor, Reproducibility, and Proportionality

Each GMMA sample was assayed in duplicate at two different dilutions that gave an absorbance within the range of the standard curve. Each sample (GMMA or BSA) gives a characteristic color yield in each assay: the absorbance per mass of protein (as measured by AA quantification). Comparing the absorbance of the GMMA samples to the BSA standard curve, we initially express GMMA protein content as “BSA equivalent” i.e., the amount (µg) of BSA that gives the same absorbance as the GMMA sample. Then for each combination of sample and assay we define the “color yield factor” as the color yield of BSA/color yield of GMMA in that assay. In practice, this was calculated by dividing the protein content determined by quantitative AA analysis, by the BSA equivalent. Thus, for subsequent assays, multiplying the “BSA equivalent” by the “color yield factor” gives the GMMA protein quantity.

The assays were performed independently three times (for *Neisseria* and *Salmonella* GMMA) or five times (for *Shigella* GMMA) and the inter-assay variation was calculated as the coefficient of variance in percent (CV %) from the mean BSA equivalent determined in each assay and the corresponding standard deviation of the results. Intra-assay variation was calculated for each experiment from the 4 measurements performed in one assay. The intra-assay variation of multiple independent assays was expressed as the mean of the intra-assay variations determined in each assay. For all the assays, the measurements of GMMA concentration were performed in the linear range of the BSA standard curve.

Data over the measurement range were fitted to a non-weighted linear regression, and the deviation of the predicted Y axis intercept from zero was used as a measure of the proportionality of the assay.

### SDS-PAGE

GMMA were denatured for 10 min at 95 °C in sodium dodecyl sulfate–polyacrylamide gel electrophoresis (SDS-PAGE) sample buffer containing 2 % (wt/vol) SDS and subsequently loaded onto 12 % (wt/vol) polyacrylamide gels (Invitrogen). Gels were run in 3-(*N*-morpholino)-propanesulfonic acid (MOPS) buffer (BioRad) and were stained with brilliant Blue G-colloidal Coomassie (Sigma-Aldrich B2025) according to the manufacturer’s instructions with slight modifications. Briefly, after 30 min fixation with 40 % (vol/vol) methanol and 7 % (vol/vol) acetic acid in water, gels were stained overnight using 40 mL of reconstituted brilliant Blue G-colloidal Concentrate (0.1 % (wt/vol) Brilliant Blue G, 0.29 M phosphoric acid, 16 % saturated ammonium sulfate) and 15 mL of methanol. Gels were destained using 30 % methanol in water (vol/vol) for 2 h.

## Results

### Color Yield and Reproducibility

The protein quantification results for different GMMA using AA quantification, Bradford, Lowry, and NI assays, and the reproducibility of the assays are listed in Table 2. This table reports the results obtained by the colorimetric assays of GMMA in comparison to the absorption of the BSA standard curve (BSA equivalents). The BSA equivalents and the results of the quantitative AA analysis were then used to calculate the color yield factor for each assay and GMMA combination.

**Table 2:** Protein quantification results of GMMA by Bradford, Lowry, Non-Interfering assay (NI), and amino acid quantification

GMMA from:	Amino acid quantification (μg/mL)	BSA equivalent^a^ (μg/mL)					Mean (μg/mL)	Standard deviation (µg/mL)	CV %^b^ inter-assay	CV %^b^ intra-assay	Color yield factor
		1	2	3	4	5					
Bradford assay											
*Ss* −p ΔOAg	14,200	3,700	3,190	3,747	4,858	4,795	4,058	735	18.1	12.7	3.50
*Ss* +p ΔOAg	5,080	1,250	1,177	1,445	1,554	1,893	1,464	283	19.3	13.9	3.47
*Ss* +p	7,910	1,650	2,086	2,129	2,375	1,951	2,038	266	13.0	11.7	3.88
*Sf*2a +p ΔOAg	8,310	3,247	2,691	2,969	2,790	3,413	3,022	304	10.1	7.6	2.75
*Sf*2a +p	5,090	2,690	2,432	2,455	2,631	2,344	2,510	145	5.8	14.2	2.03
*Sf*2a −p ΔOAg	8,510	1,545	1,298	1,718	1,880	2,258	1,740	361	20.8	14.2	4.89
*Salmonella*	8,170	1,805	2,097	2,186			2,029	200	9.8	7.0	4.03
*Neisseria*	656	338	399	476			404	69	17.1	11.3	1.62
Lowry assay											
*Ss* −p ΔOAg	14,200	17,024	16,309	20,844	18,043	20,630	18,570	2,073	11.2	11.2	0.76
*Ss* +p ΔOAg	5,080	6,774	5,973	7,872	6,861	6,759	6,848	676	9.9	9.9	0.74
*Ss* +p	7,910	9,758	10,650	11,405	11,911	11,681	11,081	879	7.9	5.3	0.71
*Sf2a* +p ΔOAg	8,310	10,748	10,546	12,182	10,278	12,113	11,173	905	8.1	6.5	0.74
*Sf2a* +p	5,090	7,667	7,488	8,101	6,387	7,555	7,440	635	8.5	5.9	0.68
*Sf2a* −p ΔOAg	8,510	11,350	12,490	12,221	10,385	11,681	11,625	825	7.1	8.6	0.73
*Salmonella*	8,170	11,415	10,500	10,360			10,758	573	5.3	7.2	0.76
*Neisseria*	656	1,136	1,265	1,205			1,202	65	5.4	11.4	0.55
NI assay											
*Ss* −p ΔOAg	14,200	17,580	19,615	20,297	19,942	17,863	19,059	1,249	6.6	7.1	0.75
*Ss* +p ΔOAg	5,080	3,090	5,448	3,463	7,015	6,159	5,035	1,704	33.8	20.4	1.01
*Ss* +p	7,910	7,950	9,755	9,911	10,995	10,648	9,852	1,180	12.0	9.9	0.80
*Sf2a* +p ΔOAg	8,310	9,690	11,454	11,610	11,172	11,254	11,036	772	7.0	10.0	0.75
*Sf2a* +p	5,090	3,400	4,627	5,412	5,221	4,849	4,702	790	16.8	18.5	1.08
*Sf2a* −p ΔOAg	8,510	10,930	12,692	11,577	11,453	10,805	11,491	748	6.5	7.2	0.74
*Salmonella*	8,170	8,287	9,182	9,060			8,843	485	5.5	12.7	0.92
*Neisseria*	656	901	880	837			873	33	3.7	9.8	0.75

Values shown under BSA equivalents 1–5 are the results of 5 (*Shigella*) or 3 (*Salmonella, Neisseria*) independent experiments of the colorimetric assays, each representing the mean of 4 repetitions in the assay

^a^BSA equivalent describes the yield of the colorimetric assays compared to the amount of BSA that would give the same color using a BSA standard curve run with each assay

^b^CV % is the coefficient of variation (ratio of the standard deviation to the mean) expressed in percent

**Fig. 1:**
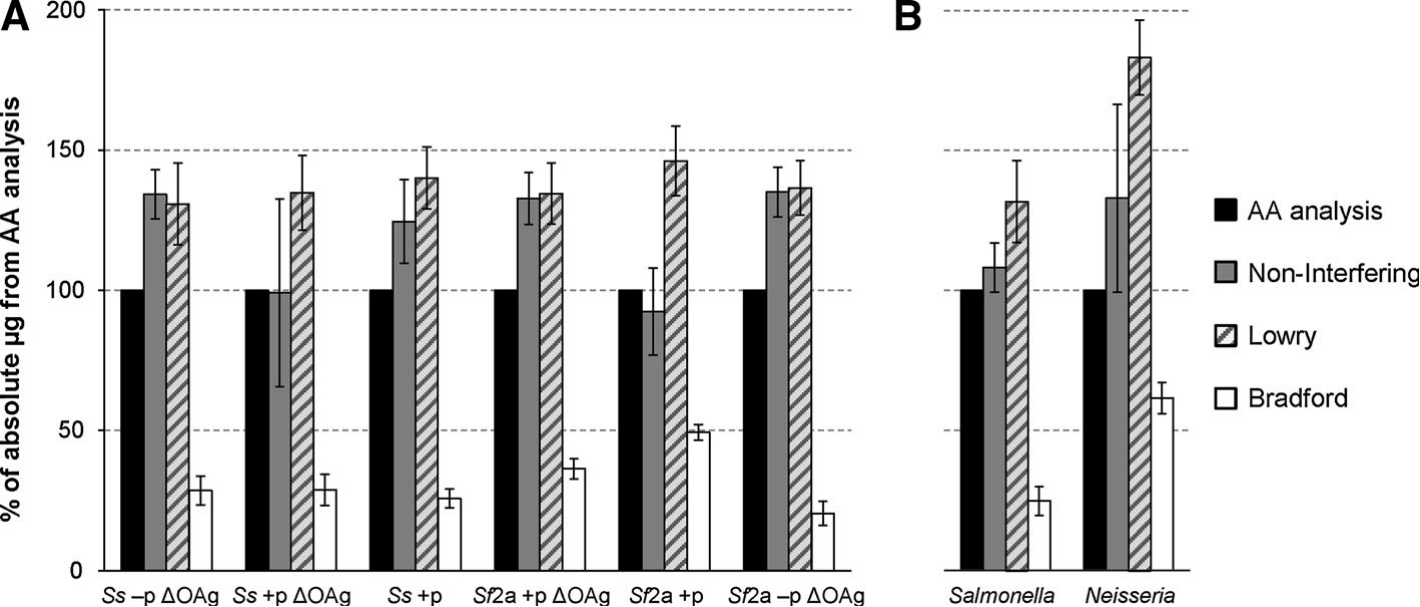
Protein quantification results of GMMA by colorimetric assays relative to AA quantification. BSA equivalents obtained by the colorimetric assay and assay standard deviations (Table 2) of GMMA from **a** *Shigella* mutants *Ss* −p ΔOAg, *Ss* +p ΔOAg, *Ss* +p, *Sf*2a +p ΔOAg, *Sf*2a +p, *Sf*2a −p ΔOAg, and **b** *Salmonella* or *Neisseria mutants*, were normalized to the respective protein concentration obtained by AA quantification. *Columns* represent the mean BSA equivalent of at least 3 independent measurements

For all of the GMMA, the Bradford assay gave a substantially lower color yield than for the equivalent amount of BSA. Therefore, if the BSA standard is used to estimate GMMA protein without applying a color yield factor, the Bradford assay will underestimate the protein content by up to 80 % (Fig. 1). In addition, the difference between the Bradford results and the quantitative AA analysis results varied for GMMA produced from different species and with different genetic modifications, without a clear correlation to the phenotypic background. For example, in multiple assays, GMMA from *Shigella* *Sf*2a +p ΔOAg consistently had nearly twice the BSA equivalent (i.e., half the color yield factor, Table 2) as *Sf*2a −p ΔOAg although they have similar AA compositions (Table 3) and were tested at similar concentrations as determined by AA analysis.

**Table 3:** Amino acid composition of GMMA from different strains

	*Sf*2a −p ΔOAg		*Sf*2a +p		*Sf*2a +p ΔOAg		*Ss* −p		*Ss* +p ΔOAg		*Ss* +p		*Salmonella*		*Neisseria*	
	nmol/mL	%	nmol/mL	%	nmol/mL	%	nmol/mL	%	nmol/mL	%	nmol/mL	%	nmol/mL	%	nmol/mL	%
Cysteic acid	–		–		–		–		–		–		–		–	
Hydroxyproline	–		–		–		–		–		–		–		–	
Aspartic acid	13,600	17.5	7,970	17.0	12,800	16.6	20,900	15.9	7,210	15.3	11,500	15.8	11,500	14.9	704	11.5
Threonine	4,860	6.3	2,810	6.0	4,550	5.9	8,960	6.8	3,100	6.6	4,910	6.8	5,190	6.7	349	5.7
Serine	4,910	6.3	3,270	7.0	5,440	7.1	8,000	6.1	2,900	6.7	4,420	6.1	4,840	6.3	457	7.5
Glutamic acid	6,960	9.0	4,620	9.8	7,330	9.5	12,300	9.4	4,050	8.6	6,350	8.7	6,800	8.8	663	10.8
Proline	1,750	2.3	1,270	2.7	2,080	2.7	3,940	3.0	1,260	2.7	2,130	2.9	2,500	3.2	157	2.6
Glycine	7,280	9.4	4,950	10.5	8,110	10.5	16,000	12.2	5,980	12.7	7,890	10.9	11,500	14.9	695	11.4
Alanine	7,290	9.4	4,290	9.1	7,410	9.6	11,200	8.5	3,970	8.4	6,280	8.7	6,820	8.8	656	10.7
Cystine	50.5	0.1	0	0	49.3	0.1	119	0.1	34	0.1	58.8	0.1	0	0	0	0
Valine	5,090	6.6	2,690	5.7	4,730	6.1	8,440	6.4	2,970	6.3	4,710	6.5	4,440	5.76	511	8.4
Methionine	624	0.8	504	1.1	582	0.8	775	0.6	228	0.5	790	1.1	575	0.75	27.7	0.5
Isoleucine	2,530	3.3	1,470	3.1	2,620	3.4	4,820	3.7	1,720	3.6	2,660	3.7	2,490	3.23	194	3.2
Leucine	5,470	7.0	3,470	7.4	5,370	7.0	8,760	6.7	3,120	6.6	4,710	6.5	4,640	6.02	313	5.1
Tyrosine	3,870	5.0	2,150	4.6	3,410	4.4	6,650	5.1	2,330	4.9	3,630	5.0	4,100	5.32	277	4.5
Phenylalanine	2,810	3.6	1,550	3.3	2,410	3.1	4,800	3.7	1,700	3.6	2,660	3.7	2,800	3.63	213	3.5
Histidine	2,420	3.1	1,310	2.8	1,890	2.5	2,910	2.2	1,940	4.1	3,080	4.2	1,160	1.50	163	2.7
Tryptophan	–		–		–		–		–		–		–		–	
Lysine	4,490	5.8	2,550	5.4	4,510	5.9	7,560	5.8	2,660	5.6	4,110	5.7	4,990	6.47	450	7.4
Arginine	3,600	4.6	2,040	4.3	3,540	4.6	5,250	4.0	1,920	4.1	2,760	3.8	2,840	3.68	285	4.7
Total	77,600	100	46,900	100	76,900	100	131,000	100	47,100	100	72,600	100	77,100	100	6,110	100

In contrast, the Lowry assay gave a higher color yield than BSA. Thus, in this case, if the BSA standard is used without applying a color yield factor, then this will lead to overestimation of the protein content of the GMMA, for *Shigella* and *Salmonella* GMMA consistently by about 35 % (Fig. 1). *Neisseria* GMMA gave a lower color yield factor than *Shigella* and *Salmonella* GMMA for both the Bradford and Lowry assays (Table 2). The *Neisseria* GMMA have a different AA distribution compared to the *Shigella* and *Salmonella* GMMA (Table 3).

With the NI assay, GMMA gave on average approximately 20 % higher color yield than BSA resulting in an overestimation of the protein content (Table 2; Fig. 1). However, there was variation between closely related GMMA, e.g., the *Sf*2a +p ΔOAg gave a 30 % lower color yield factor than *Sf*2a +p.

In addition to being the most consistent assay for different types of GMMA (CV % of the color yield factors for *Shigella* GMMA were 3.5, 16.0, and 26.1 for Lowry, NI, and Bradford assays, respectively), the Lowry assay gave a lower average inter- and intra-assay variation compared with the other methods (Table 2). With quartz cuvettes, the reproducibility of the Lowry assay was further improved compared to using plastic cuvettes: the intra-assay variation decreased from an average of 9 % (*Shigella* GMMA) to less than 4 % for each of the tested samples (Table 4).

**Table 4:** Intra-assay variation by Lowry assay using quartz cuvettes

GMMA from:	Amino acid quantification (μg/mL)	BSA equivalent^a^ (μg/mL)				Mean (μg/mL)	Standard deviation (µg/mL)	CV^b^ % intra-assay
		1	2	3	4			
*Ss* −p ΔOAg	14,200	21,064	20,001	19,353	19,885	20,076	717	3.6
*Ss* +p ΔOAg	5,080	6,657	6,976	6,826	6,454	6,728	225	3.3
*Sf*2a +p ΔOAg	8,310	11,449	11,272	10,845	10,491	11,014	431	3.9
*Sf*2a −p ΔOAg	8,510	11,256	11,522	11,293	11,718	11,477	215	1.9

Values shown under BSA equivalents 1-4 represent 4 individual repetitions performed within one assay

^a^BSA equivalent describes the yield of the colorimetric assays compared to the amount of BSA that would give the same color using a BSA standard curve run with each assay

^b^CV % is the coefficient of variation (ratio of the standard deviation to the mean) expressed in percent

### Proportionality

**Fig. 2:**
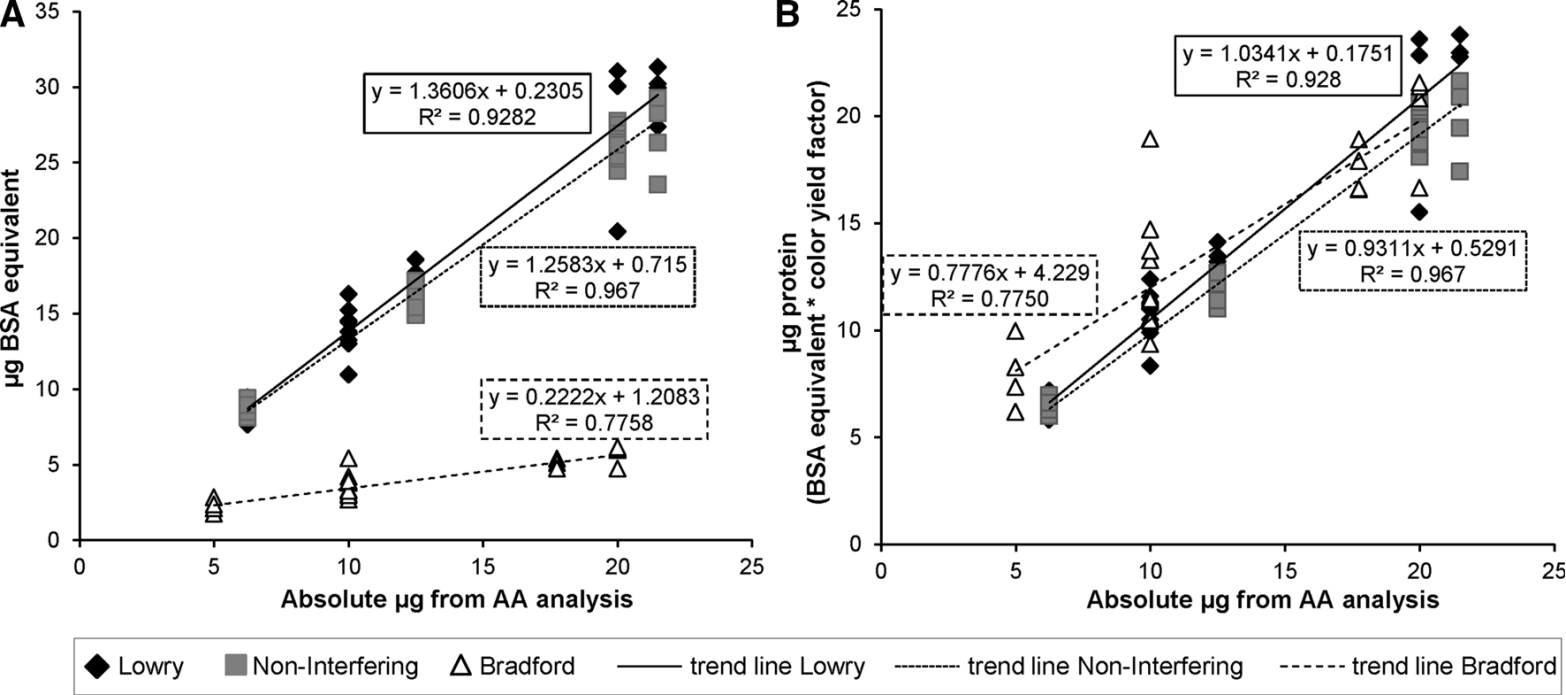
Linearity and proportionality of assays to results by AA quantification. GMMA purified from *Ss* −p ΔOAg were assayed using Bradford, Lowry, and NI assay in the range of 5.0–20.0 µg (Bradford) and 6.25–21.5 µg (Lowry and NI assay) of absolute protein as quantified by AA analysis. **a** BSA equivalents as obtained from the assays or **b** protein amounts calculated from the BSA equivalents multiplied by the color yield factors for each assay (3.5, 0.76, and 0.74, respectively, for Bradford, Lowry, and NI assay, see Table 2) are plotted against the absolute protein amount measured by AA analysis. Linear regression lines and regression parameters for each assay were calculated and shown on graphs

*Ss* −p ΔOAg GMMA were assayed in the range of 5.0–20.0 µg protein (AA quantification) by Bradford assay and at 6.25–21.5 µg by Lowry and NI assay to compare the increase of protein quantity as measured by the assay with the increase of absolute protein amount according to AA quantification. Two different comparisons were performed. First, in the absence of a color yield factor, i.e., as would be the case for new types of GMMA that have not yet been analyzed by AA quantification, BSA equivalents obtained from the assays were plotted against the absolute protein amount measured by AA analysis and analyzed by linear regression (Fig. 2a). Second, for GMMA with a known color yield factor, protein amounts calculated from the BSA equivalents multiplied by the color yield factors for each assay were similarly analyzed (Fig. 2b). The criteria for evaluating the trend lines obtained for each colorimetric assay were the slope, intercept, and the ‘goodness of fit’ (*R* ^2^ value). The assay performance is better the closer the slope is to 1 (a measure of the equivalence of the increase of the measured protein quantity to AA analysis results), the intercept to 0 (a measure of proportionality of response to AA analysis results), and the *R* ^2^ value to 1 (a measure of the reproducibility of the assay). In both analyses, of the three assays, Lowry gave the results closest to the above criteria (formulas reported in Fig. 2a, b). Results from the NI assay were generally similar to the Lowry with a slightly bigger intercept. Bradford results gave a lower slope and higher intercept. The *R* ^2^ value of trend line was lower for Bradford than for Lowry and NI assay.

### Evaluation of Equivalence of Results by SDS-PAGE

**Fig. 3:**
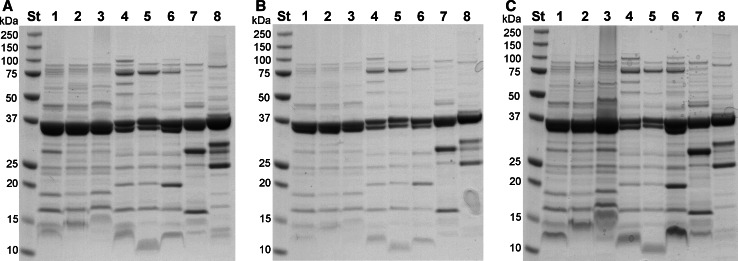
Semi-quantitative analysis of GMMA by SDS-PAGE. GMMA from 1. *Ss* −p ΔOAg, 2. *Ss* +p ΔOAg, 3. *Ss* +p, 4. *Sf*2a +p ΔOAg, 5. *Sf*2a +p, 6. *Sf*2a −p ΔOAg, 7. *Salmonella*, and 8. *Neisseria* were analyzed by 12 % SDS-PAGE using the following quantities/well: **a** 10 μg protein as measured by AA quantification, **b** 10 µg BSA equivalent as determined by Lowry, and **c** 2.5 µg BSA equivalent as obtained by Bradford. Gels were stained with Coomassie Blue

Since the color yields of different proteins vary [11], the protein compositions in the different GMMA could bias the average protein quantification. Thus, the protein quantification results obtained by Lowry and Bradford methods were assessed semi-quantitatively by analyzing GMMA by SDS-PAGE (Fig. 3). As the NI assay uses a similar chemistry to the Lowry assay, a separate analysis was not performed. Samples were loaded at 10 µg total protein by AA analysis (Fig. 3a), at 10 µg BSA equivalents by Lowry (Fig. 3b, corresponding to 6.8–7.6 µg *Shigella*, 7.6 µg *Salmonella,* and 5.5 µg *Neisseria* GMMA protein after applying the respective color yield factors in Table 2) or at 2.5 µg BSA equivalents by Bradford (Fig. 3c, corresponding to 5.1–12.2 µg *Shigella*, 10.0 µg *Salmonella*, and 4.1 µg *Neisseria* GMMA protein). All three loading conditions gave similar results.

## Discussion

We have examined several factors to determine the most useful in-process assay for measuring the protein concentration of GMMA from different bacteria. Those factors are: a combination of ease, reproducibility and proportionality of the assay, and the comparability of the results for different GMMA.

We compared Bradford, Lowry, and NI assays using a BSA standard calibrated against GMMA concentrations determined by AA analysis. By the above criteria, the Lowry assay was superior. First, it was the simplest assay to perform, requiring neither a strong chaotropic agent (guanidine) and boiling (Bradford) or multiple centrifugations (NI assay) and took significantly less time to perform than the other assays: approximately 15 min for Lowry compared to approximately 45 min for Bradford and 35 min for the NI assay. Second, the Lowry assay also gave substantially lower inter- and intra-assay variation compared with the other two assays. Third, the Lowry assay gave consistent results for GMMA from different genotypes whereas Bradford and NI assay showed substantial variability between GMMA with similar composition. Both, Lowry and NI assays gave good proportionality and linearity (as measured by linear regression) over a range of protein concentrations useful for vaccine production and testing (around 5–25 µg) with the Lowry assay being slightly superior to the NI assay.

For all three assays, and particularly the Bradford assay, the color yield was substantially different to the color yield of the equivalent measured amount of BSA in the same assay. This highlights the necessity of calibrating a secondary standard such as BSA against a primary GMMA standard quantified by AA concentration. The resulting color yield factor is applied to convert the results of the calorimetric assay (BSA equivalent) to absolute protein amounts as determined by AA analysis. Without applying the color yield factor, the Bradford assay substantially underestimated the amount of protein present and the Lowry modestly overestimated the protein concentration. While the color yield factors generally varied between GMMA from different bacteria within the same genus, the differences were larger between different bacterial families, with the *Neisseria* GMMA showing lower color yield factors for both the Bradford and Lowry assays in accordance with a different AA distribution compared to the other tested GMMA from *Shigella* and *Salmonella*.

The NI assay relies on depletion of copper ions in solution when they bind to peptide bonds and generally has minimal protein to protein variation. Using the NI assays performed in our studies, the color yield for GMMA was on average closest to the color yield of BSA. However, there was substantial variation between different GMMA, greater intra- and inter-assay variation and it is a more complicated assay to perform than the Lowry assay. In the absence of a primary GMMA standard, for many applications these considerations are likely to outweigh the small overestimation in GMMA protein concentration that would result from the use of the Lowry assay calibrated only against a BSA standard. In general, the Lowry assay has been reported to show better results than other assays for membrane-containing fractions [20], together with a low inter-protein variability [11], and thus is beneficial for analyses when the standard is a different protein from the measured sample [11].

Finally, these results highlight the need for caution when comparing results from different laboratories if different protein assays are used and if they have not been calibrated using a primary GMMA standard that has been quantified by AA analysis. In the absence of such calibration, GMMA assayed by Bradford and Lowry, two commonly used methods, would have given more than a fivefold difference in the apparent protein concentrations: an error sufficiently large to impact on interpretation of reactogenicity and immunogenicity of GMMA-based and potentially other candidate vaccines.
